# Spectroscopic Assessment of Platinum Group Elements of PM_10_ Particles Sampled in Three Different Areas in Jeddah, Saudi Arabia

**DOI:** 10.3390/ijerph17093339

**Published:** 2020-05-11

**Authors:** Mohammad W. Kadi, Iqbal Ismail, Nadeem Ali, Abdallah A. Shaltout

**Affiliations:** 1 Department of Chemistry, Faculty of Science, King Abdulaziz University, P.O. Box 80203, Jeddah 21589, Saudi Arabia; iqbal30@hotmail.com; 2 Center of Excellence in Environmental Studies, King Abdulaziz University, Jeddah 21589, Saudi Arabia; nabahadar@kau.edu.sa; 3 Spectroscopy Department, Physics Division, National Research Centre, Cairo, El Behooth St., 12622 Dokki, Egypt; shaltout_a@hotmail.com; 4 Physics Department, Faculty of Science, Taif University, P.O. Box 888, Taif 21974, Saudi Arabia

**Keywords:** ICP-MS, PM_10_ aerosols, platinum group elements (PGE), Jeddah

## Abstract

Platinum group elements (PGE) including Ru, Rh, Pt and Pd have been quantified in air particulate matter with an aerodynamic diameter equal or less than 10 microns (PM_10_) using inductively coupled plasma mass spectrometry (ICP-MS). PM_10_ aerosols have been collected from three sites representing various activities in Jeddah city, Saudi Arabia. These locations are residential site with heavy traffic, industrial site and heavy traffic and a light traffic site outside the city. To obtain reasonable data of the PGE concentrations, a group from 10 to 15 PM_10_ samples were collected every month. The annual and seasonal variation of the mass concentration of the PGE were demonstrated. In all locations, Pt and Pd were relatively higher than Ru and Rh possibly because their main use is in automobile catalytic converters. Concentrations of observed PGE in PM_10_ could be arranged in ascending order as: Rh < Ru < Pd < Pt. In case of Ru and Pt, there are clear similarities in terms of the overall mean concentrations at the sampling locations. Due to the high concentration of Ru, Rh and Pd at low traffic site, there are certainly other sources of these elements rather than vehicle catalytic converters. However, at the industrial/heavy traffic location, high concentrations of Ru were detected during February 2015. In addition, high Pt concentrations were also detected at the light traffic site during May 2015. Results indicate that Pt source in PM_10_ is mainly the automobile catalytic converters.

## 1. Introduction

In the last decades, notable increase in the production of the platinum group elements, including platinum (Pt), palladium (Pd), rhodium (Rh) and ruthenium (Ru), has been recognized. Although platinum group elements (PGE) have low natural abundance in the continental crust, they have a wide range of applications in electrical, chemical, pesticide, dyestuff production, polymer processing, pharmaceuticals (anticancer drugs, hydration and dehydration reactions), dental implants, and industrial applications including petroleum industries [1]. In the years of 1975 and 1986, the PGE were introduced as automobile exhaust catalysts in North America and Europe, respectively [1]. The objective was to convert noxious gases produced by the vehicles’ exhaust, such as nitrous oxide, carbon monoxide, and various hydrocarbons emissions into amiable forms. Afterwards, the use of PGE as automobile exhaust catalysts was widespread all over the world. Prior to the application of vehicle exhaust catalysts, the concentrations of the PGE were less than 1 pg m^−3^ or almost not found in the ambient air. Nowadays, the industrial application of automobile catalytic converters consumes a great portion of the PGE production [2,3]. For noxious gas reduction, Pt and Pd were mainly used along with Rh at various ratios. As the PGE are extensively used to decrease noxious gases in air, their concentrations in ambient air are rising over time with a dependence on several parameters such as; type of engine, type of catalyst, traffic density and speed of the driving [4,5]. Although air quality improves as a result of the introduction of PGE as catalytic converters, a remarkable increase in the concentration of these metals in the atmosphere was detected [6,7,8,9,10,11]. Therefore, the increase of PGE in the atmosphere may cause further environmental contamination especially at specific locations such as garages, tunnels and high traffic-density areas.

Due to the low concentrations of the PGE in ambient particulate matter, there is limited information about their toxicity, environmental bioavailability and influence on human health [12,13]. In the presence of chloride, halogenated PGE complexes might be produced, which have damaging influence on lung cells. Using Te and Hg co-precipitation methods, the concentration of the PGE (Pt, Pd, and Rh) has been determined in fractionated airborne dust, PM_10_, PM_2.5_ and PM_1_ collected from rural and urban areas in Germany [9]. In all size fractions, the concentration of the PGE increased over time, and remarkable high concentration of Pd was found compared to Pt and Rh. The highest concentration of PGE was found in PM_10_, and its concentration decreased as the size of the fractionated airborne particles decreased [9,14]. An increase in the concentration of PGE levels in urine as a function of exposure by bus drivers and traffic police officers has been demonstrated [15,16]. The determination of PGE concentration in PM_10_ collected from different cities around the world has been presented in the literature [1,6,7,8,9,10,11,17,18,19,20,21]. Although PGE are not soluble in water, their toxicity and chemical forms depend on their solubility in the biological media [1,3,22].

Several studies have presented concentrations of PGE in airborne particulate matters collected from different locations in Europe, Latin America, United States, Canada and China [5,6,7,9,10,17,21,23,24,25]. However, information about concentration levels of PGE in airborne particulate matters collected from Saudi Arabia is not available. Therefore, the present work represents the first attempt to determine the PGE in PM_10_ samples collected from three different locations in Jeddah city, Saudi Arabia. PM_10_ aerosols were collected during the period from June 2014 to May 2015 covering the four seasons of the year. The three sampling locations have different activities in terms of traffic intensity, populations and industrial activities. Gathering information about the concentration and the spatial distribution of the PGE during the working day, weekend and the overall annual mean in PM_10_ samples is the aim of the present work. Besides, seasonal variations of PGE were demonstrated.

## 2. Materials and Methods

### 2.1. Reagents and Materials

Deionized water with a resistance of 18.2 MΩ cm was obtained from a Milli-Q system (Millipore, Billerica, MA USA) and used throughout. Multi element standard solutions of Au, Hf, Ir, Pd, Rt, Rh, Ru, Sb, Sn and Te were used and prepared freshly by proper dilution from 10 mg L^−1^ stock standard solution (Perkin Elmer, Waltham, MA, Germany). Nitric acid (69%, Merck, Darmstadt, Germany) and hydrochloric acid (37%, Merck, Darmstadt, Germany) were used for PM_10_ digestion. A certified reference material (CRM) of atmospheric particulate matter (NIST 1648a, National Institute of Standard and Technology, Gaithersburg, ML, USA) was used to confirm the accuracy of the present analytical procedure. The CRM was digested with the same digestion procedure of PM_10_ samples.

### 2.2. Sampling Collection

Three sequential air samplers (Partisol Plus 2025-D, Thermo Fisher Scientific, Waltham, MA, USA) were used to simultaneously collect PM_10_ samples from three different locations at a height of ~10 m above the ground and far away from the direct influence of the traffic density. Quartz microfiber filters with a diameter of 47 mm were used for PM_10_ collection. Filters were weighed before and after sampling to determine the mass concentrations of the PM_10_ samples. The filters were weighed using a micro-balance (Model XPE206DR, Mettler Toledo, Muntinlupa, Philippines). The air sampler can hold up to 16 filter sets and can be programmed to automatically change the filters according to a pre-set sampling periods. Samples were collected for twelve-month period starting June 2014 through May 2015 covering the different four seasons. Each sample was collected every other day for 24 h at flow rate of 15 L min^−1^ with the following environmental parameters recorded for each sample: sampling time, atmospheric temperature, atmospheric pressure, sampling flow rate, sampling volume, wind speed and relative humidity. Therefore, the number of collected samples was about 180 samples/location and a grand total of PM_10_ samples was 545 samples.

**Figure 1:**
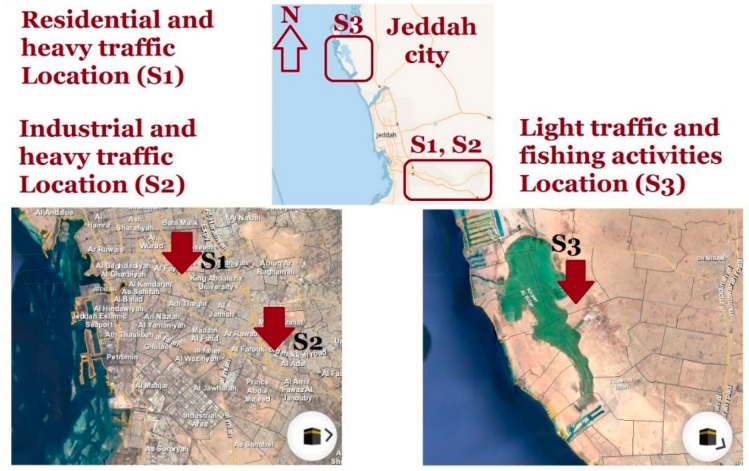
Air particulate matter with an aerodynamic diameter equal or less than 10 microns (PM_10_) sampling locations in Jeddah city, Saudi Arabia.

PM_10_ samples were collected from three different locations in Jeddah city, Saudi Arabia, which have different environmental conditions in terms of traffic density, human activities and population. The first location was the main campus of King Abdulaziz University (S1) with a latitude of 21.49 and longitude of 39.23. It is characterized as a residential/heavy traffic location, whereas the location represents a populous region near to the major highway. Besides, the location has a heavy traffic not only on the nearby highway, but also around the campus of the university. More than 70,000 faculty members and students are usually using their private vehicles as means of transportation. The second location is located at the southeast of the city at the old Makkah road (S2) and has a latitude of 21.45 and longitude of 39.29. It has heavy duty traffic with heavy trucking dominated. In addition, the second location has many industrial activities such as different types of workshops, ceramic tiles fabrication, construction materials and many other light industries. The last location is called Durrat Alarous (S3), which is a beach resort, and it has a latitude of 21.9 and longitude of 39.02. The location is on the red sea shore and it is characterized with low traffic and sparse population. The positions of the three sampling locations in Jeddah is illustrated in Figure 1.

### 2.3. Digestion of the PM_10_ Samples

Collected PM_10_ samples on the quartz microfiber filters were digested completely using a microwave digestion system (TOP wave Analytik Jena AG, Jena, Germany). Aqua regia (12 mL; 3 mL of 69% HNO_3_ and 9 mL of 37% HCl) was used for digestion. The digested PM_10_ samples were diluted to a final volume of 25 mL with deionized water.

### 2.4. Inductively Coupled Plasma Mass Spectrometry (ICP-MS)

An inductively coupled plasma mass spectrometer ELAN 6000 (NexION 300D, Perkin Elmer, Waltham, MA, USA) was used for the quantitative analysis of the PGE in the PM_10_ particulates. Pure argon gas was used as a plasma gas and for nebulization at flow rate of 0.65 L min^−1^. A microconcentric nebulizer (MCN−100, CETAC Technologies, Omaha, USA) coupled with a cyclonic spray chamber was used. For the sample flow control, a peristaltic pump (Ismatec, Glattbrugg, Switzerland) was used. Before using the microconcentric nebulizer, performance checks for sensitivity of oxide and doubly charged ions formation, using a conventional cross flow nebulizer and a Scott spray chamber was carried out. The digested PM_10_ aerosols were injected into the nebulizer with a flow rate of 30 μL min^−1^. The radio frequency (RF) power was 1600 W. The lens voltage, analog stage voltage and the pulse stage voltage were 9.55 V, −1745 V, and 950 V, respectively. The instrument conditions were auto lens mode on, peak hopping measure mode, dwell time of 40 ms, 40 sweeps/reading, reading/replicate, 3 replicates, and 1200 ms integration time. Platinum sampler and skimmer cones were used.

### 2.5. Quality Assurance of the Analytical Method

Quality assurance was checked through the analysis of a CRM. Recoveries were in agreement with the certified values which indicate good analytical values of the observed concentrations. Table 1 illustrates the measured and certified concentrations of the CRM 1648a, which is composed of atmospheric particulate matter. Unfortunately, there is no certified values for the PGEs.

## 3. Results and Discussion

### 3.1. Quantitative Elemental Analysis of Platinum Group Elements

**Figure 2:**
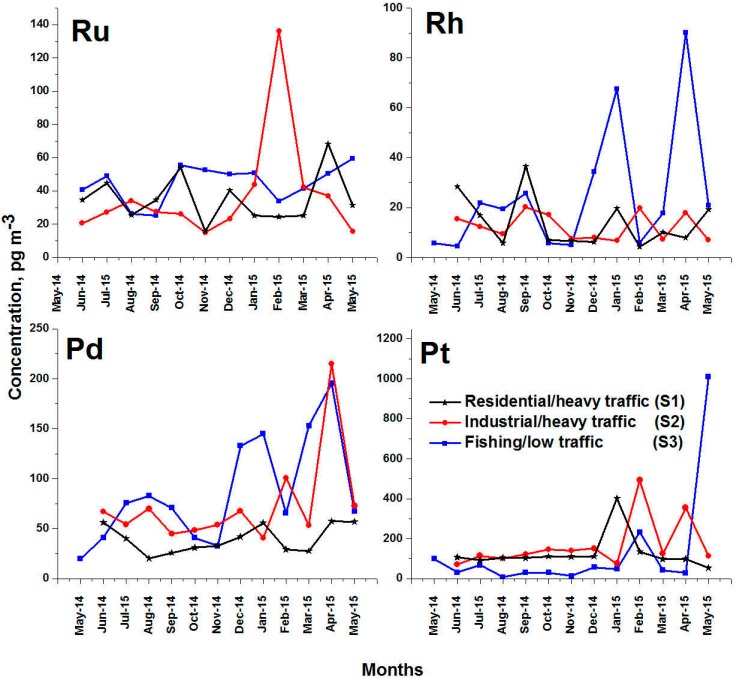
Overall monthly averages of platinum group elements (PGE) detected in PM_10_ from the three sampling sites in Jeddah city.

Standard calibration curves starting from 0 to 7000 ng/L were prepared and used for the ICP-MS. An excellent linearity of the calibration curves was obtained with regression coefficients range from 0.998 to 0.999. Although there are six PGE (Ru, Rh, Pd, Pt, Os and Ir), the concentrations of Os and Ir are not detectable in the ambient air particulates using the ICP-MS. In addition, Os and Ir were never reported in the air particulates. The quantitative analysis results were calculated in pg/m^3^. For the current three locations, the monthly averages of the detected concentration of the PGE in PM_10_ aerosols is illustrated in Figure 2. During each month, approximately fifteen PM_10_ samples were collected. The concentrations of the PGE in some PM_10_ aerosols were undetectable as they fall below the limits of detection. Therefore, the monthly averages represent the mean values of the detected PGE in the collected PM_10_ aerosols for the month. The annual mean concentrations and the ranges of the PGE are illustrated in Table 2.

In the case of Ru, the concentration variations at sites S1, S2, and S3 were 15.9 ± 2.1–68.3 ± 1.1 pg m^−3^, 15.1 ± 0–136.3 ± 194.6, 25.3 ± 1.5–59.6 ± 0, respectively. The overall mean concentrations of Ru at sites S1, S2 and S3 were 35.4 ± 14.6 pg m^−3^, 37.4 ± 32.5 pg m^−3^, 43.3 ± 11.8 pg m^−3^, respectively. Although there is a variation of Ru concentration from one location to another, the overall mean concentrations in all locations have clear similarities. Distinguishable high Ru concentration was also detected at site S2 and its reached 136.3 ± 194.6 pg m^−3^ during February 2015. As the production of Ru increased recently [26], additional applications of Ru were also found, e.g., as wear resistant electrical contacts, thick film resistors, and ultraviolet photomasks. In addition, it is used in mixed metal oxide anodes that are used for cathodic protection of underground and submerged structures. The industrial wide range of Ru applications could be the main source in the atmosphere.

Concentrations of Rh in PM_10_ sample are always lower than Ru. The monthly average concentrations of Rh at sites S1, S2 and S3 were 14.0 ± 10.2 pg m^−3^, 12.4 ± 5.4 pg m^−3^, and 24.9 ± 26.1 pg m^−3^, respectively. At S1 and S2 sites which are recognized as heavy traffic/residential and heavy traffic/industrial, the variations of Rh were 4.4–36.5 pg m^−3^ and 7.6–20.2 pg m^−3^, respectively; small variations were observed at S1 and S2 locations, and the Rh concentrations were always less than the 40 pg m^−3^ detected during September 2014 at the S1 location. At S3 location, which is recognized as a light traffic, Rh concentrations have remarkable variation, ranging from 5.0 to 90.1 pg m^−3^. The largest variation of Rh concentration was due to the high concentration of Rh during individual days of January and April 2015, which reached to 430.1 pg m^−3^. It is clear that, location S3 has the highest concentration of Rh, which is approximately twice the detected values in S1 and S2 locations. The high value of standard deviation at S3 locations (~26 pg m^−3^) indicates the instability of the atmospheric aerosol near to the red sea coast. Although S3 location is recognized as a low traffic site, the high concentration of Rh originates from the automobile catalytic converters due to the high atmospheric dispersion during January and April 2015. As it is well known, approximately 80% of the produced Rh is used as catalysts. Additional sources of Rh could be the fiber glass and flat-panel glass industry, as well as the high temperature and corrosion resistive materials [27].

Looking to Pd, its concentrations in PM_10_ aerosols collected from different sites is relatively higher than Ru and Rh but lower than Pt. The overall monthly average concentrations of Pd were 39.5 ± 13.8 pg m^−3^, 86.3 ± 53.6 pg m^−3^ and 73.9 ± 47.2 pg m^−3^, at sites S1, S2 and S3, respectively. Although the S3 location was considered as low traffic, its Pd concentration during April represents the highest values comparing with other locations. Besides, the variations of Pd concentration at S3 site ranges from 19.6 ± 24.9 to 195.3 ± 200.4 pg m^−3^. At locations S1 and S2, the variations of Pd concentrations were 20.3 ± 16.4 to 57.2 ± 42.7 pg m^−3^ and 40.5 ± 27.2 to 214.9 ± 589.7 pg m^−3^, respectively. Starting from December 2014 up to May 2015, there was an increase in Pd concentration at sites S2 and S3 whereas the overall concentration of Pd at the university site (S1) had relatively minor changes. The main sources of Pd in the atmosphere could be the heavy usage of Pd in vehicle catalytic converters, jewelry, blood sugar test strips, dentistry, and electric contacts [28]. Of course the vehicle catalytic converters represent the main source of Pd, but the additional mentioned sources of Pd could participate in PM_10_ aerosols. This could be deuced from the relative higher concentrations of these elements at the low traffic site (S3).

Pt represents the main element in the PGE, and its concentration in all locations represents the highest concentration comparing with other PGE. The monthly overall average concentrations of Pt were 126.4 ± 88.6 pg m^−3^, 166.9 ± 126 and 130.1 ± 271 pg m^−3^, at locations S1, S2 and S3, respectively. As shown in Table 2, the overall averages of the detected concentrations of Pt in all sites are relatively close to each other, which is very interesting. This could be reasonable because Pt was the main element used for automobile catalyst converters. Pt could be released during operation of the vehicle due to the mechanical deterioration and abrasion. The emission of PGEs is depending on the operating condition of the engine, the type of the catalysts, the type of the fuel additives and the vehicle speed. However, the variation of Pt concentrations in all sites was also recognized. At location S1, the Pt varies from 52.6 ± 74.5 to 401.3 ± 1100 pg m^−3^, and its concentration during January 2015 was relatively high. At location S2, Pt varies from 70.3 ± 40.4 to 492.5 ± 1015 pg m^−3^, and the highest Pt concentration was found during February and April 2015. At location S3, Pt varies from 6.5 ± 0 to 1009.4 ± 126 6 pg m^−3^, and the highest Pt concentration was detected during May 2015. The variation of the concentration of Pt at the low traffic area (S3) is lower than in other areas of heavy traffic, even though the average values are comparable, which confirms the source of the Pt is in air, Figure 2. In addition, the average concentration of Pt at heavy traffic/industrial area represents the highest value, which refers to an additional source of the Pt in the atmosphere, which is the industrial activities.

### 3.2. Annual Mean Values of PGE

Based on the individual measurements during the period of study and regardless of the seasonal variation as well as working days and weekends, the annual mean values for each element was calculated and is presented in Table 2. As expected, the highest concentration of the PGE belongs to Pt followed by Pd and then Ru and finally Rh. The annual mean values of Ru and Rh are comparable in locations S1 and S2 whereas the highest annual mean value was measured at location S3. In the case of Pd, the lowest concentration was found at the residential/ heavy traffic site (S1) whereas its concentration increases at the industrial/heavy traffic area (S2). The highest concentration of Pd was found at the fishing /low traffic area (S3). In the case of Pt, the industrial/heavy traffic area (S2). The highest concentration of Pd was found at the industrial/heavy traffic area (S2) whereas Pt concentration in the two other sites is comparable. The high contents of PGE at the location S2 (industrial/heavy traffic) confirms the existence of other industrial sources rather than the automobile catalytic converters.

### 3.3. Seasonal Variations of PGE

**Figure 3:**
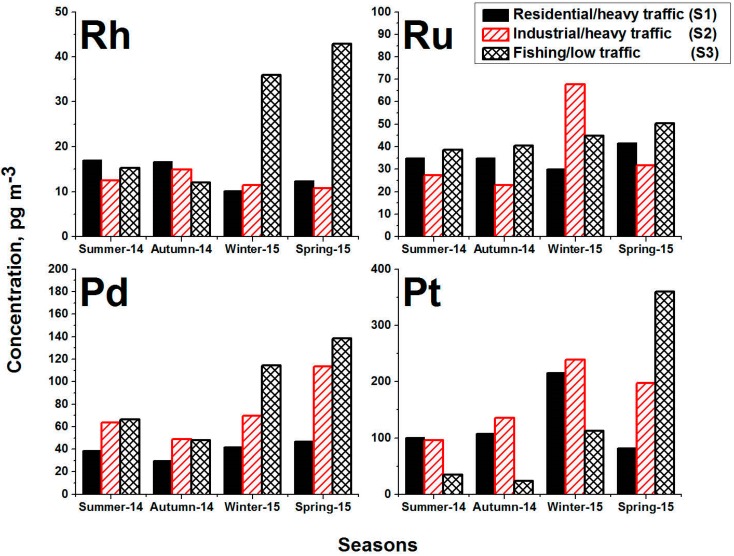
Seasonal variations of Ru, Rh, Pd and Pt in PM_10_ collected from three different sites in Jeddah city.

The seasonal variations of Ru, Rh, Pd and Pt during the four seasons of the sampling period are illustrated in Figure 3. The overall average of each season is presented. In the case of Rh, the average concentrations at the sites S1 and S2 have a recognized low variation, and these values fluctuate from 10 ± 8 to 17 ± 11 pg m^−3^, Figure 3. The remarkable stability of Ru concentrations at the two locations (S1 and S2) during all four seasons confirms the association of the sources of Rh, which is the vehicle catalytic convertors. The low contents of Rh could be an indication of its low contents in the catalytic convertors. The case is different at the low traffic site (S3) whereas high concentrations of Rh were found with high standard deviation values especially during winter and spring 2015. The maximum Rh concentration at location S3 was found during the spring 2015 (43 ± 141 pg/m^3^). The high concentration of Ru during the winter and spring 2015 at location S3 could be an indication of the low dispersion in the atmosphere during these seasons as well as other anthropogenic sources. Besides, ship emissions may be an additional source because the location S3 is close to the international Sea Port of Jeddah. In case of Ru, there is a clear stability of the concentrations at the three sites with few exceptions. A slight increase of Ru during winter and spring 2015 at location S3 was recognized. Furthermore, a remarkable increase of Ru at location S2 during winter with remarkable high standard deviation was also found. The use of Rh and Ru for catalytic converters is generally very limited, this could be the reason for the stability of Rh and Ru concentrations at all locations. Possible sources of the Rh and Ru in the atmosphere could be the natural resources in the surrounding desert and anthropogenic sources including catalytic converters, aircrafts, glass and polymer production [25].

Looking at the distribution of Pd concentrations, higher concentrations were observed during winter and spring at all sites with noticeable large standard deviations. The maximum seasonal value was found at location S3 during spring 2015, and it equals 138 ± 65 pg m^−3^. The lower seasonal values were found during the summer and autumn 2014. Similar seasonal behavior was also observed for Pt whereas the highest Pt concentrations were found during the winter and spring seasons with high standard deviation. The highest concentration of Pt was found at location S3 and it equals 360 ± 563 pg m^−3^. As the Pt and Pd are the main elements used for the automobile catalytic convertors, their concentrations in the ambient air particulates are always higher than Ru and Rh. Previous studies reported that the concentration of various elements increased during winter due to the low dispersion of atmospheric aerosols, which causes the increase in the concentrations at locations that have high traffic [29,30,31,32,33]. The same manner was found not only during the winter but also during the spring 2015, Figure 3. The low variations of the PGE could be recognized in most of the three locations during the summer and autumn seasons whereas high variations were found during the winter and spring seasons. At the heavy traffic/residential area (S1), there are no remarkable seasonal changes in the concentration of Ru, Rh and Pd, and the variations are in the range of the standard deviation values. The heavy traffic/industrial area (S2) has higher concentrations of Pt and Pd than the location S1 during all seasons. On the other hand, the low traffic site (S3) had the highest concentrations of PGE during spring 2015.

### 3.4. Concentrations of PGE during Working Days and Weekends

**Figure 4:**
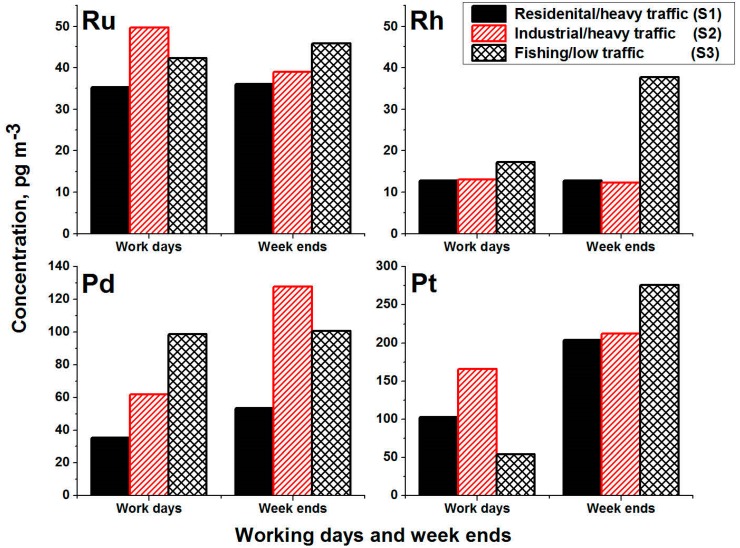
Concentration variations of Ru, Rh, Pd and Pt during working days and weekends in PM_10_ collected from three different sites in Jeddah city.

To illustrate the variation of the PGE concentration during working days and weekends, Figure 4 depicts the annual averages during the whole year for Ru, Rh, Pd, and Pt. The use of the annual mean values of the PGEs concentrations, as an indication of the variation during working days and weekends, gives an acceptable estimation of these elements in Jeddah’s atmosphere. Generally, there is a clear indication of the increase of PGE concentrations during weekends with the exception of Ru concentration at location S2. During the weekends, concentrations of Pd increased at the heavy traffic areas (S1 and S2), and no changes of concentration were recognized at the low traffic area (S3). However, the industrial/heavy traffic location (S2) had higher concentrations of PGE than the residential/heavy traffic location (S1) during the working days and weekends. Therefore, sources of PGE at the industrial/heavy traffic area (S2) are not only automobile catalytic converters but also other industrial and anthropogenic sources. The high concentration of the PGE during the weekends at locations S1, S2 and S3 could be an indication of the low dispersion in the atmosphere during the weekends. In addition, the noticeable increase of the number of vehicles and trips coming to Jeddah city during the weekends might be an indication of the increase of the exhausts from the vehicles in the atmosphere. Due to the potential use of PGE in automobile catalytic converters, there are risks to humans and the environment because the emission of PGE in the atmosphere is a combination between chemical and thermal aging, which is easily solubilized and mobilized in the atmosphere. In addition, the fine PGE emission uptake by the organism could result in conversion to toxic species associated with increases of mortality [23].

### 3.5. Comparison with Published Data

Table 3 illustrates a comparison between the observed quantitative analysis results of the PGE and the published data for PM_10_ aerosols. The comparison was carried out for the available published data from the following cities; Göteborg, Sweden; Madrid, Spain; Rome, Italy; Frankfurt, Germany; Buenos Aires, Argentina; Vienna, Austria; Boston, USA; Mexico City, Mexico. Determination of Ru in PM_10_ aerosols was presented only in the PM_10_ samples of the present work, and it was not quantified in the PM_10_ aerosols all over the world earlier. The other PGE were compared with the different mentioned locations. In the case of Rh, the observed results in the three sites in Jeddah city show somewhat elevated values [7].

A similar case was also found for Pd where observed concentrations are higher than those reported in the literature (Rome, Italy) [7]. The case of Pt in the present work is relatively similar to Pd. The obtained average value of Pt (130.1 ± 271 pg m^−3^) is higher than the highest values found in the literature at Frankfort, Germany, which is equal 15.7 pg m^−3^ [14]. It is worthwhile to mention that averages observed here are from limited numbers of sample and not extensive sampling each day of the year. However, the obtained averages of the PGE could represent the annual mean values whereas the sampling was continued for a whole year from June 2014 to May 2015. Concentrations of PGE in Jeddah city are characterized by high concentrations compared with the published data. This could be due to several reasons. In Jeddah city, the inhabitants are basically using their private automobiles for transportation since public transport is very scarce. In addition, the high concentration of PGE in Jeddah’s atmosphere could indicate the absence of recycling of spent catalytic converters, which may result in erosion of their material due to environmental factors and hence escape to the atmosphere. This is of course in addition to their use in operating vehicles. Furthermore, the main reason for the presence of PGE could be its heavy use in the automobile catalytic converter and the dental PGE alloys, given that at Jeddah city, the basic mean of transportation is private vehicles and trucking. It was mentioned that the number of vehicles in Jeddah city reached 1.5 Million [32]. In addition, PGE and its compositions are usually used as a chemical modifier in graphite furnace atomic absorption spectrometry [33,34,35,36,37,38], which may increase the concentration of PGE in the ambient air.

## 4. Conclusions

This work presents the first quantitative elemental analysis of PGE in the PM_10_ aerosols collected from three different sites that have low and heavy density of traffic in Jeddah, Saudi Arabia. For the first time, Ru quantification in the present PM_10_ aerosols was determined whereas it was not quantified before in the PM_10_ aerosols all over the world. The observed levels of PGEs in Jeddah show higher concentrations than reported values possibly because the shear high numbers of operating vehicles and the lack of recycling of spent catalytic converters in the city. Higher concentrations at a site with low traffic density could indicate that catalytic converters are not the only source of these elements. In case of Pt, the average concentrations in all locations are more or less similar, which confirms the same origins in the catalytic converters. The noticeable increase of the PGE concentrations at the industrial/heavy traffic locations confirms the contribution of the industrial activities beside the catalytic converter source.

## Figures and Tables

**Table 1:** Certified and measured concentration of the detected elements in the certified reference material (SRM 1678a).

Element	Certified Values	Measured Values	Unit
As	115.5 ± 3.9	111.55 ± 6.3	µg/g
Cd	73.7 ± 2.3	70.76 ± 4.5	µg/g
Ce	54.6 ± 2.2	22.68 ± 3.4	µg/g
Co	17.93 ± 0.68	14.50 ± 1.1	µg/g
Cr	402 ± 13	387.97 ± 33	µg/g
Cu	610 ± 70	763 ± 54	µg/g
Fe	3.92 ± 0.21	3.5 ± 0.11	%
Mg	0.813 ± 0.012	0.74 ± 0.021	%
Mn	790 ± 44	703.74 ± 56	µg/g
Ni	81.1 ± 6.8	88.1 ± 7.6	µg/g
Pb	0.655 ± 0.033	0.63 ± 0.041	%
Sb	45.4 ± 1.4	41 ± 2.7	µg/g
V	127 ± 11	86 ± 17	µg/g
Zn	4800 ± 270	4790 ± 321	µg/g

**Table 2:** The annual mean values and the ranges of PGE in PM_10_ at the three sites in Jeddah.

El.	Concentration, pg m^−3^		
	Location (S1)	Location (S2)	Location (S3)
Ru	35.4 ± 14.6 (15.9 − 68.3)	37.4 ± 32.5 (15.1 − 136.3)	43.3 ± 11.8 (25.3 − 59.5)
Rh	14.0 ± 10.2 (4.4 − 36.5)	12.4 ± 5.4 (7.0 − 20.2)	24.9 ± 26.1 (4.5 − 90.1)
Pd	39.5 ± 13.8 (20.3 − 57.2)	74.0 ± 47.2 (40.5 − 214.9)	86.3 ± 53.6 (19.6 − 195.3)
Pt	126.4 ± 88.6 (52.6 − 401.3)	166.9 ± 126 (70.3 − 492.5)	130.1 ± 270.5 (6.5 − 1009.4)

**Table 3:** Concentration of the PGE in PM_10_ (pg m^−3^) collected from various cities.

Reference	Location/Conditions	City/Country	Concentration, pg/m^3^			
			Ru	Rh	Pd	Pt
Present work	Low traffic/fishing (S3)	Jeddah, Saudi Arabia	43.3 ± 11.8 (25.3 − 59.5)	24.9 ± 26.1 (4.5 − 90.1)	86.3 ± 53.6 (19.6 − 195.3)	130.1 ± 270.5 (6.5 − 1009.4)
Present work	Heavy traffic/industrial (S2)	Jeddah, Saudi Arabia	37.4 ± 32.5 (15.1 − 136.3)	12.4 ± 5.4 (7.0 − 20.2)	74.0 ± 47.2 (44.8 − 214.9)	74.0 ± 47.2 (44.8 − 214.9)
Present work	Heavy traffic/residential (S1)	Jeddah, Saudi Arabia	35.4 ± 14.6 (15.9 − 68.3)	14.0 ± 10.2 (4.4 − 36.5)	39.5 ± 13.8 (20.3 − 57.2)	126.4 ± 88.6 (52.6 − 401.3)
[8]	70,000 cars/day	Göteborg, Sweden	-	2.9 (1.3 − 4.3)	4.9 (1.3 − 9.7)	14.1 (7.6 − 19.2)
[8]	<10,000 cars/day	Göteborg, Sweden	-	0.6 (0.3 − 1.2)	1.8 (< 0.6 − 4.4)	2.1 (0.9 − 3.0)
[6]	Five sites			3.3 (< 0.2 − 12.2)		12.8 ( < 0.1 − 57.1)
[7]	Downtown	Madrid, Spain		2.8		7.3
[7]	Ring-road	Madrid, Spain		4.6		17.7
[7]	Downtown	Göteborg, Sweden		2.7	4.6	13.1
[7]	Ring-road	Göteborg, Sweden		0.8	1.6	4.1
[7]	Downtown	Rome, Italy	-	2.2	42.7	8.6
[7]	Ring-road	Rome, Italy		3.0	54.9	8.1
[14]	Major street (32550 cars/day)	Frankfurt, Germany		2.9 (1.8 − 4.5)	25.1(9.4 − 29.3)	15.7(8.7 − 28.4)
[14]	Side Street (< 1000 cars/day)	Frankfurt, Germany		0.7 (0.7–1.1)	8.9 (5.1–15.6)	6.2 (4.1–9.5)
[14]	nonurban	Frankfurt, Germany		0.8 (0.3–1.5)	7.8 (4.7–11.7)	5.2 (3.0–7.9)
[2]	Seven inner city sites	Buenos Aires, Argentina		3.9 (0.3 − 16.8)		12.9 (2.3 − 47.7)
[20]	Downtown	Vienna, Austria		0.4 ± 0.1	2.6 ± 0.6	4.3 ± 1.7
[19]	Site 1 (20000 cars/day)	Boston, USA		2.2 (0.5–5.9)	11.0 (1.0–26)	9.4 (0.6–17)
[19]	Site 2 (10000 cars/day)	Boston, USA		1.3 (0.3–5.9)	7.1 (0.8–39)	6.2 (0.6–36)
[18]	Five sites	Mexico City, Mexico		3.2 ± 2.2	11 ± 4	9.3 ± 1.9
